# The Action of Key Factors in Protein Evolution at High Temporal Resolution

**DOI:** 10.1371/journal.pone.0004821

**Published:** 2009-03-12

**Authors:** Armin Schmitt, Johannes Schuchhardt, Gudrun A. Brockmann

**Affiliations:** 1 Institute for Animal Sciences, Humboldt-Universität zu Berlin, Berlin, Germany; 2 MicroDiscovery G. m. b. H., Berlin, Germany; 3 Interdisciplinary Center for Variability and Adaptation, Humboldt-Universität zu Berlin, Berlin, Germany; Cardiff University, United Kingdom

## Abstract

**Background:**

Protein evolution is particularly shaped by the conservation of the amino acids' physico-chemical properties and the structure of the genetic code. While conservation is the result of negative selection against proteins with reduced functionality, the codon sequences determine the stochastic aspect of amino acid exchanges. Thus far, it is known that the genetic code is the dominant factor if little time has elapsed since the divergence of one gene into two, but physico-chemical forces gain importance at greater evolutionary distances. Further details, however, on how the influence of these factors varies with time are unknown to date.

**Methodology/Principal Findings:**

Here, we derive each 10,000 divergence specific substitution matrices for orthologues and paralogues from the Pfam collection of multiple protein alignments and quantify the action of three physico-chemical forces and of the structure of the genetic code at high resolution using correlation analysis. For closely related proteins, the codon sequence similarity is the most influential factor controlling protein evolution, but its influence decreases rapidly as divergence grows. From a protein sequence divergence of about 20 percent on the maintenance of the hydrophobic character of an amino acid is the most influential factor. All factors lose importance from about 40 percent divergence on. This suggests that the original protein structure often does no longer represent a constraint to the protein sequence. The proteins then become free to adopt new functions. We furthermore show that the constraints exerted by both physico-chemical forces and by the genetic code are quite comparable for orthologues and paralogues, however somewhat weaker for paralogues than for orthologues in weakly or moderately diverged proteins.

**Conclusion/Significance:**

Our analysis substantiates earlier findings that protein evolution is mainly governed by the structure of the genetic code in the early phase after divergence and by the conservation of physico-chemical properties at the later phase. We determine the level of sequence divergence from which on the conservation of the hydrophobic character is gaining importance over the genetic code to be 20 percent. The evolution of orthologues and paralogues is shaped by evolutionary forces in quite comparable ways.

## Introduction

The evolution of proteins can be seen as a succession of replacements of amino acids by other amino acids. In order to quantify the rates by which amino acids are replaced by other amino acids so-called substitution matrices (or exchange matrices) are built from multiple sequence alignments of homologous proteins [1]. Substitution matrices are of particular importance for sequence data base searches with protein or DNA sequences of unknown function. Many attempts were made to refine them [2], [3], [4]. Such a substitution matrix is, strictly speaking, specific for the protein it is derived from because not all positions in a protein are of equal importance. Furthermore, substitution matrices describing the amino acid exchanges between strongly diverged proteins differ from those that describe amino acid exchanges between weakly diverged proteins [5]. We can thus speak of a time dependence of substitution matrices under the assumption that the metaphor of the molecular clock is essentially valid [6].

Several attempts were made to understand the changeability between amino acids and, hence, the elements of substitution matrices. It has become clear that essentially two factors have to be considered: the structure of the genetic code and the conservation of an amino acid's physico-chemical character [7], [8]. The genetic code assigns each amino acid one or more codons with specific three-nucleotide-sequences. Amino acids can be coded for by as many as six different codons (L, R, and S) or by a unique codon (M and W). Exchanges between amino acids should therefore be facilitated if their codons are mostly similar in sequence, i. e. if they primarily differ by just one nucleotide [9]. Such amino acid exchanges should be relatively frequent. Conversely, exchanges between amino acids which are essentially coded for by dissimilar codons should be relatively rare.

Apart from the genetic code, an amino acid's physico-chemical properties have to be taken into account. Since substitution matrices are built from fully functional proteins it can be assumed that during an exchange one amino acid is preferably replaced by one with similar physico-chemical properties. This would more likely guarantee the functionality of the protein as a whole than replacement by a dissimilar amino acid. The tendency to remain conserved can thus be interpreted as a result of physico-chemical forces which act in such a way that replacements by dissimilar amino acids are avoided and replacements by similar amino acids are favored. Above all, the three properties hydrophobicity, polarity and volume determine an amino acid's physico-chemical character.

The relative importance of these two major evolutionary players, genetic code and conservation of physico-chemical properties, was quantified previously in protein variants from one and the same species and for closely related proteins [10]. As predicted in an earlier work [11], the genetic code is the major factor controlling evolution within species and physico-chemical forces gain importance in the protein evolution between species. In this work we are interested in studying the influence that the physiochemical factors and the structure of the genetic code exert on the amino acid exchanges as a function of the time that has elapsed since the divergence between two proteins.

To this end we first construct substitution matrices from pairwise protein alignments with a degree of sequence identity varying from 99 down to ten percent (corresponding to degrees of divergence between one and 90 percent) in steps of one percent and correlate them with the matrix of differences in volume, hydrophobicity, polarity and average codon sequence difference.

If the genetic code were the sole factor governing amino acid exchanges then those exchanges should prevail whose codon sequence distance is low. There should therefore be a strong anti-correlation between the codon distance matrix and the empirical substitution matrix. Likewise, if the physico-chemical factors were the sole factors, then those exchanges should predominate whose differences in the physico-chemical properties differ little. The correlation coefficient between the four distance matrices and the substitution matrices for the various degrees of protein sequence divergence allow therefore to estimate the relative strength of the corresponding factors for each degree of divergence.

We are also interested to correlate the changes that are involved in amino acid substitutions on the physico-chemical level and on the genetic code level. Are the physico-chemical changes correlated with each other and with codon sequence changes? A strong correlation between physico-chemical properties and genetic code properties would mean that the genetic code has protection built in against strong changes in the physico-chemical properties. It could be shown in previous work [12], [13], [14] that changes in the codons that can occur easily by chance, e. g. through a single transition, are mostly involving small changes in the physico-chemical properties. This means that the function of a protein is unlikely disrupted e. g. by occasionally occurring replication errors. In other words, some protection is already imprinted in the genetic code, and it could be shown that this protection is especially marked for hydrophobicity [15]. Here, we are interested to quantify the degree of protection that is intrinsic in the genetic code.

## Methods

### Amino acid substitution matrices

We downloaded 10,340 multiple alignments via ftp from http://pfam.sanger.ac.uk/ (file Pfam-A.full, Pfam Version 23.0, as of July, 2008 [16]) Theses protein families were constructed from 6,145,588 individual protein sequences. From each alignment we picked each two orthologous and two paralogous human sequences randomly. We determined the degree of identity between these pairs of sequences by counting the number of sites with identical amino acids and subsequent division by the total number of aligned amino acid pairs. The degree of divergence of an alignment was calculated as 100 minus the degree of identity in percent rounded to the closest integer. Insertions and deletions were not considered. The counts for each alignment were sorted into a 20×20 matrix. Matrices belonging to one and the same degree of divergence between one and 90 percent were then added such that 90 divergence specific substitution matrices resulted. We confined ourselves to one sequence pair each from a multiple alignment as a basis for the substitution matrices (instead of considering all possible pairs) in order to avoid bias towards those proteins whose multiple alignments were constructed from many sequences [2]. The raw counts in the substitution matrices were multiplied with the relative frequencies of the amino acids in the protein alignments of the corresponding degree of divergence so that the values were corrected for unequal amino acid frequency.

### Differences in three physico-chemical properties

We calculated the absolute differences in physico-chemical properties for each of the 190 possible amino acid pairs for three fundamental properties: amino acid volume, polarity and hydrophobicity based upon entry numbers GRAR740103, GRAR740102 [17], and SWER830101 [18] of the database AAindex [19]. Since we calculated absolute differences, the differences associated with the exchange of a given amino acid by another and with the reverse exchange are identical. By construction, the resulting matrices are symmetric.

### Codon sequence distance

To describe amino acid exchange frequencies in terms of the genetic code, we determine for each exchange the average distance between their codon sequences. As an example, how this measure is calculated we consider glutamic acid and glycine which can be coded for by two and four codons, respectively. An exchange between these two amino acids can thus be assigned eight codon pairs. In two cases, GAA ↔ GGA and GAG ↔ GGG, a one nucleotide swap suffices, in six cases, GAA ↔ GGC, GAA ↔ GGG, GAA ↔ GGT, GAG ↔ GGA, GAG ↔ GGC, and GAG ↔ GGT, two nucleotides have to be changed to attain the amino acid exchange. On average, the codon sequence distance between glutamic acid and glycine is 1.75 nucleotides. As with the physico-chemical changes we arranged the codon sequence distances into 20×20 matrices. The assignment of amino acids to codons was taken from the R-package seqinR [20].

### Correlation analysis

We used the function mantel (with the method kendall and with the default of 1000 permutations) from the R-package vegan [21] to calculate the correlation coefficient between the property difference matrices and the amino acid substitution matrices.

## Results

### Correlation between evolutionary factors

**Figure 1:**
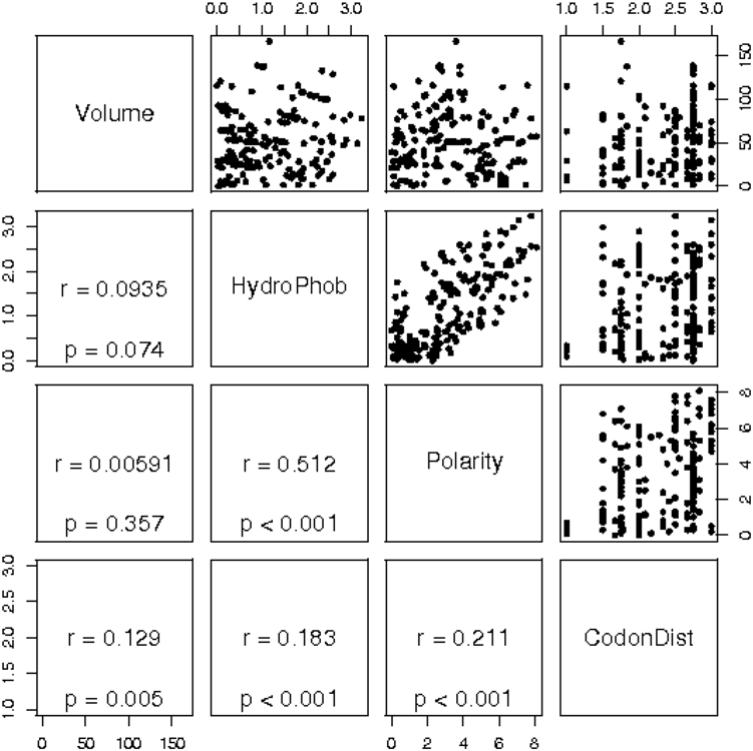
Correlation between four evolutionary factors. Shown are scatterplots for the differences in three physico-chemical properties and in the average codon sequence that are associated with the 190 possible amino acid exchanges (upper triangle of the scheme) and the corresponding correlation coefficients and p-values obtained in a Mantel test (lower triangle of the scheme). Differences in the physico-chemical properties are weakly correlated with average differences in the codon sequences. Abbreviations: HydroPhob, hydrophobicity; CodonDist, average codon distance.

First, we were interested if the four factors that influence amino acid exchanges are largely independent or if there is correlation between them. Figure 1 shows all pairwise scatter plots for the changes in volume, hydrophobicity, polarity, and mean codon distance that the 190 possible amino acid substitutions entail. The only strong correlation was found between differences in hydrophobicity and polarity (r = 0.512). Volume is nearly uncorrelated with hydrophobicity and polarity. There is also weak, however statistically highly significant, correlation between differences in the three physico-chemical properties on the one hand and the mean codon distance on the other hand. This means that the amino acids' physico-chemical conservation is partially intrinsic to the genetic code, but it cannot be explained through the genetic code alone. A marked feature of the plots hydrophobicity and polarity vs. average codon distance is that amino acid exchanges that can exclusively be realized by one nucleotide swaps entail almost constant hydrophobicity and polarity. In contrast, no marked tendency can be seen for average codon distances greater than 1.

### Amino acid substitution matrices in the course of time

We determined amino acid substitution matrices from 10,135 and 10,057 orthologous and paralogous pairwise protein sequence alignments, respectively. The number of orthologous alignments contributing to the divergence specific substitution matrices ranged between 74 and for a divergence of one percent and 27 for a divergence of 90 percent. The corresponding numbers were 462 and 21 for paralogous alignments. Degrees of divergence greater than 90 percent were not considered because too few alignments contributed to these substitution matrices. First, we studied some of these divergence specific matrices by eye. An obvious feature is that many of the exchanges are not observed at all when divergence is low. Table 1 shows the substitution matrix derived from orthologous protein alignments which are divergent at five percent of their sites. The sum of all counts was set to 10,000 and then counts were rounded. Almost half of the possible exchanges (176/400) are not observed in this case. Off-diagonal elements are typically two orders of magnitude smaller than diagonal elements, i. e. the number of amino acid sites that are identical in an alignment. As divergence increases, more and more amino acid exchange pairs are observed. At a divergence of 50 percent, all but six possible exchanges are realized (Table 2). Now, off-diagonal elements are typically only one order of magnitude smaller than diagonal elements.

**Table 1:** Exchange frequencies (sum normalized to 10,000) derived from protein alignments which are divergent at 5 percent of their sites.

	A	C	D	E	F	G	H	I	K	L	M	N	P	Q	R	S	T	V	W	Y
A	730	1	1	4	0	6	0	0	0	2	0	0	2	1	0	17	14	10	0	1
C	1	171	0	0	0	1	0	0	0	0	0	0	0	0	2	2	0	0	0	2
D	1	0	543	16	0	2	0	0	0	0	0	6	0	0	0	2	0	1	0	1
E	4	0	16	634	0	4	0	0	7	0	0	0	0	2	0	0	2	0	0	0
F	0	0	0	0	397	0	0	1	0	3	0	0	0	0	0	0	0	2	1	5
G	6	1	2	4	0	582	0	0	0	0	0	1	0	0	2	11	1	1	0	0
H	0	0	0	0	0	0	177	1	0	0	0	2	1	1	2	1	0	0	0	2
I	0	0	0	0	1	0	1	547	0	5	6	0	1	0	0	1	2	11	0	0
K	0	0	0	7	0	0	0	0	619	0	0	1	0	4	10	1	2	0	0	0
L	2	0	0	0	3	0	0	5	0	890	2	0	5	2	1	5	0	4	0	0
M	0	0	0	0	0	0	0	6	0	2	229	0	0	0	0	0	3	4	1	0
N	0	0	6	0	0	1	2	0	1	0	0	405	0	0	1	8	2	0	1	0
P	2	0	0	0	0	0	1	1	0	5	0	0	426	2	1	4	2	0	0	0
Q	1	0	0	2	0	0	1	0	4	2	0	0	2	353	3	1	1	0	0	0
R	0	2	0	0	0	2	2	0	10	1	0	1	1	3	521	1	0	0	1	0
S	17	2	2	0	0	11	1	1	1	5	0	8	4	1	1	664	5	0	0	0
T	14	0	0	2	0	1	0	2	2	0	3	2	2	1	0	5	501	2	0	0
V	10	0	1	0	2	1	0	11	0	4	4	0	0	0	0	0	2	664	0	1
W	0	0	0	0	1	0	0	0	0	0	1	1	0	0	1	0	0	0	129	0
Y	1	2	1	0	5	0	2	0	0	0	0	0	0	0	0	0	0	1	0	326

**Table 2:** Exchange frequencies (sum normalized to 10,000) derived from protein alignments which are divergent at 50 percent of their sites.

	A	C	D	E	F	G	H	I	K	L	M	N	P	Q	R	S	T	V	W	Y
A	397	10	28	26	9	46	10	21	22	26	7	16	29	21	24	70	41	45	2	4
C	10	97	2	1	4	2	0	3	2	5	0	1	0	2	3	7	6	5	0	2
D	28	2	295	70	2	21	6	2	18	5	3	33	9	16	10	24	14	6	1	3
E	26	1	70	321	2	19	6	7	34	10	2	19	16	36	23	28	20	9	1	4
F	9	4	2	2	222	3	6	15	3	44	8	2	2	2	3	8	3	17	5	39
G	46	2	21	19	3	410	6	1	15	4	2	16	9	13	14	30	14	5	1	3
H	10	0	6	6	6	6	100	3	8	6	1	15	5	13	13	8	4	3	1	14
I	21	3	2	7	15	1	3	265	6	82	24	2	4	4	7	9	17	115	1	3
K	22	2	18	34	3	15	8	6	261	12	3	24	11	30	72	19	21	11	0	4
L	26	5	5	10	44	4	6	82	12	563	48	6	9	10	13	16	22	71	4	11
M	7	0	3	2	8	2	1	24	3	48	104	2	2	2	5	5	8	15	1	2
N	16	1	33	19	2	16	15	2	24	6	2	166	7	18	13	25	18	3	0	4
P	29	0	9	16	2	9	5	4	11	9	2	7	286	10	10	20	15	11	1	1
Q	21	2	16	36	2	13	13	4	30	10	2	18	10	137	22	14	11	8	2	3
R	24	3	10	23	3	14	13	7	72	13	5	13	10	22	307	18	12	11	2	4
S	70	7	24	28	8	30	8	9	19	16	5	25	20	14	18	230	56	12	2	5
T	41	6	14	20	3	14	4	17	21	22	8	18	15	11	12	56	227	33	1	4
V	45	5	6	9	17	5	3	115	11	71	15	3	11	8	11	12	33	311	1	5
W	2	0	1	1	5	1	1	1	0	4	1	0	1	2	2	2	1	1	96	7
Y	4	2	3	4	39	3	14	3	4	11	2	4	1	3	4	5	4	5	7	212

**Figure 2:**
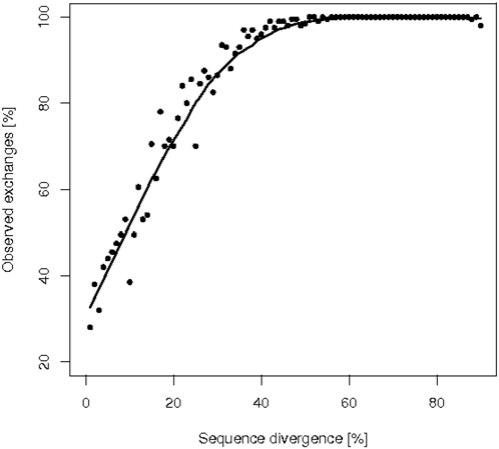
Observed exchanges. Shown is the percentage of observed amino acid exchanges in orthologes as a function of the degree of protein sequence divergence in percent of amino acid sites. The solid line is obtained by smoothing the data; it serves as guide to the eye.

**Figure 3:**
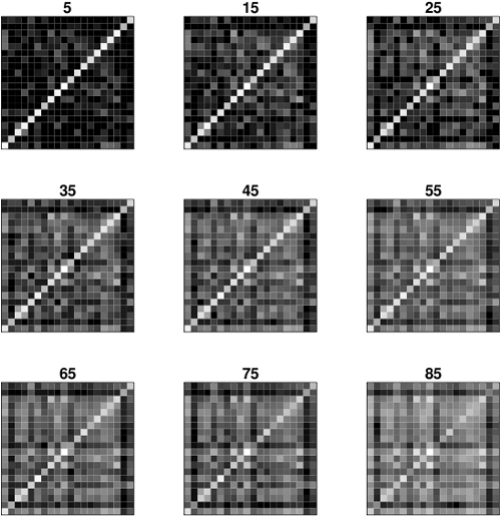
Visualized amino acid exchanges frequencies. The plots show the occupation of the nine substitution matrices which were derived from orthologous proteins diverged between five and 85 percent of their sites. The degree of divergence is given on top of each plot. Light squares indicate high occupation numbers, dark squares low occupation numbers. The amino acids are presented in alphabetical order according to their one-letter-code.

Figure 2 shows that the proportion of realized amino acid exchanges out of all possible exchanges rises fast from about 30 percent for weakly divergent proteins to in general 100 percent for orthologous proteins diverged by 50 percent or more of all sites. In order to visualize how the occupation of the substitution matrices varies with divergence we plotted the logarithm of their elements (increased by 1 to avoid negative values) in a gray scale (Figure 3). The 400 possible amino acid pairs are represented by little squares. Bright squares reflect high numbers, dark squares small numbers. As can be seen clearly, the diagonal elements vanish more and more as divergence grows, but a weak trace remains even at a divergence level of 85 percent.

### Evolutionary factors in the course of time

**Figure 4:**
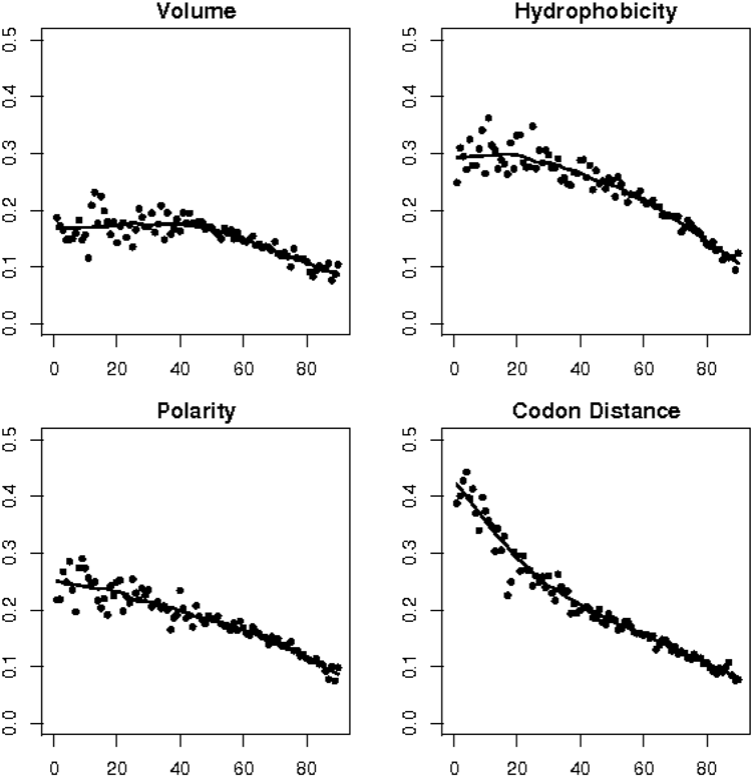
The strength of four factors controlling amino acid exchange as a function of protein sequence divergence. The curves show how the influence of the amino acids' volume, hydrophobicity, polarity and average codon sequence differences varies with the protein sequence divergence. Each dot represents the correlation coefficient (y-axis) for a substitution matrix with a property difference matrix for a certain degree of protein sequence divergence (x-axis). The correlation coefficients are negative, but are presented as absolute values for clarity's sake. The solid lines are obtained by smoothing the data; they serve as guide to the eye.

Figure 4 shows how the correlation strength between property difference matrices and the substitution matrix for orthologues varies with sequence divergence. The correlation coefficients are negative, but for clarity's sake we present their absolute values. Essentially, the influence of all four factors is decreasing with the exception of plateaux or even slight increases for volume between 0 and 40 percent and for hydrophobicity between 0 and 20 percent.

**Figure 5 pone-0004821-g005:**
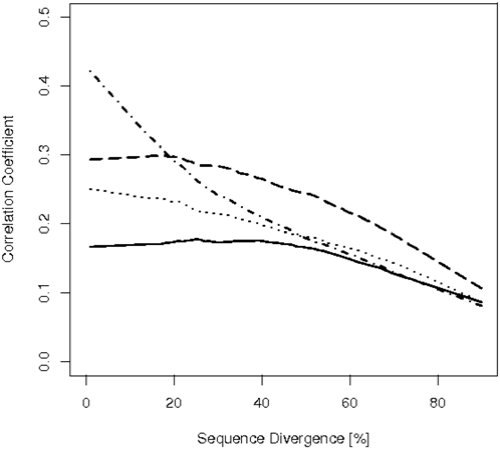
What explains amino acid exchanges best? The curves from Figure 4 are shown again in this plot to facilitate comparison. Sequence divergence is presented on the x-axis, the correlation coefficient on the y-axis. Solid line: volume; dotted line: polarity; dashed line: hydrophobicity; dot dashed line: mean codon distance. The mean codon sequence distance represents the strongest influences on amino acid exchanges when protein sequence divergence is low; the conservation of an amino acid's hydrophobicity is the prevailing factor when sequence divergence is greater than 20 percent.

In order to assess better the relative strengths of the individual factors we present their smoothed curves together in Figure 5. The dominating factor for weekly divergent proteins (up to 20 percent divergence) is the genetic code (dot dashes). For sequences diverged by more than 20 percent, the conservation of hydrophobicity (long dashes) is the most dominant factor. The conservation of volume (solid line) is the weakest force up to a divergence of 60 percent, when its curve merges with that of the genetic code. The conservation of polarity (dotted curve) plays an intermediate role. The correlation for all factors falls until 0.1 for sequences which are 90 percent divergent. We also determined the average correlation coefficient for shuffled alignments of orthologous proteins to assess if this value of 0.1 is beyond noise level. Values between 0.009 for polarity and 0.025 for the mean codon distance suggest that this is indeed the case.

**Figure 6:**
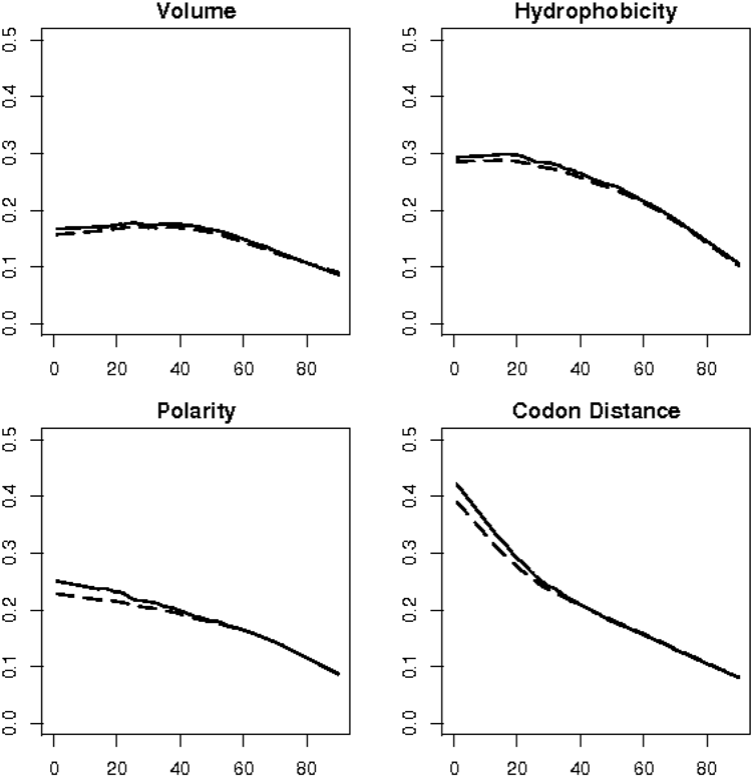
The four factors controlling amino acid exchange acting on orthologues and paralogues. The curves for orthologues from Figure 4 are shown again as solid lines, those for paralogues are shown as long dashes. The four factors exert less constraint on paralogous than on orthologous proteins when sequence divergence is low or moderate. From 50 percent divergence on the difference vanishes.

We performed the same correlation analysis for paralogues and show the results in Figure 6. Essentially, we obtained identical curve shapes for paralogues (dashed lines) as for orthologues (solid lines). An important difference, however, is that the correlation is somewhat weaker for paralogues for weakly to moderately divergent sequences. When the divergence has exceeded 50 percent, this difference vanishes practically. The difference seems to be more marked for polarity and codon distance than for volume and hydrophobicity (0.03 vs 0.01 for weakly divergent sequences).

## Discussion

After a speciation event or a gene duplication event the two copies of a gene evolve separately and their nucleotide sequences start to diverge. This divergence is in general reflected in their protein sequences. On a molecular level, the sequence divergence is caused by replacements of amino acids by other amino acids. These exchanges are not random, but are essentially constrained by the conservation of an amino acid's physico-chemical character to guarantee its functioning. At the same time, the structure of the genetic code facilitates some amino acid exchanges because they can be attained by the swap of just one nucleotide and makes others more difficult because two or three nucleotides have to be exchanged.

It has been known that protection against great amino acid alterations is a feature of the genetic code, i. e. amino acid exchanges that can be mediated by just one nucleotide swap entail mostly slight changes in an amino acid's physico-chemical character and those that are mediated by three nucleotide swaps entail mostly greater changes. Though being statistically significant, we could show that this correlation is only moderate; there are many exceptions to the rule. For example, the interchange between the hydrophilic amino acide serine (S) and the hydrophobic amino acid phenylalanine (F) can be mediated through the one-transition exchanges. TCC ↔ TTC and TCT ↔ TTT. It must also be taken into consideration that further protection could be provided by uneven codon usage, i.e. in case of multiple codons for one amino acid those codons are preferred that are dissimilar to codons that code for dissimilar amino acids. Furthermore, protection could also be provided by different exchange rates for transitions (nucleotide interchanges between purines or between pyrimidines) and transversions (nucleotide interchanges between purines and pyrimidines).

The main objective of this study was to analyze which amino acids are preferably replaced by which other amino acid depending on the degree of divergence between the protein sequences and to identify and quantify four factors controlling these exchanges.

To this end we constructed a series of each 90 substitution matrices from pairwise alignments of orthologous and of paralogous protein sequences which we excerpted from the Pfam collection of protein multiple sequence alignments. These matrices are worthwhile studying themselves. Their most marked feature is that they are poorly populated if divergence is weak. Only about half of the possible exchanges are observed, and their rate is very low. From a sequence divergence rate of 50 percent on all possible exchanges are in general observed. To quantify the strength of the influence that is exerted by the conservation of the three physico-chemical amino acid features polarity, volume and hydrophobicity, we constructed matrices that contain the differences of these quantities for each amino acid pair. To describe the differences between amino acids in terms of the genetic code we determined the codon sequence difference for each codon pair that can be associated with an amino acid exchange and averaged over all sequence distances to yield the mean codon distance.

To determine both the variation with sequence divergence and the relative strength of the four factors we correlated the four distance matrices with the 90 substitution matrices. Interpreting these correlation strengths as influence that is exerted by the four factors it can be concluded that immediately after the divergence of two proteins the structure of the genetic code is the predominant factor controlling amino acid substitutions. It is in this regime above all the ability to realize an amino acid exchange with the exchange of just one nucleotide that is the decisive factor to explain the empirical exchange rates. The conservation of an amino acid's hydrophobicity plays the leading role when sequence divergence has grown beyond 20 percent. Beyond 50 percent sequence divergence, the strengths of polarity, volume and mean codon distance are comparable.

It has been known since Zuckerkandl's pioneering work that new functions can be performed once proteins have diverged [22], but it has remained obscure if this is the rule or the exception. In [23] it is suggested that the adoption of new functions is the rule for paralogues, but it is the exception for orthologues, at least if there is no gene duplication after speciation (1 ∶ 1 homology). Despite possibly new functions for the derived proteins that can be quite different from the original function similar constraints are exerted onto paralogues and orthologues. Here, we have shown that the conservation of an amino acid's physico-chemical character exerts less and less constraint as divergence proceeds. In analogy to the physico-chemical forces, the genetic code is the underlying structure that facilitates exchanges between amino acids whose codon sequences are similar and makes those exchanges harder whose codon sequences are dissimilar. However, this “guiding force” is also losing influence as protein sequence diverges.

Thus, the proteins become less and less bound to their original sequence permitting them to adopt other conformations and to fulfill additional or new functions. The adoption of new functions seems to be a gradual process. We could furthermore show that, at least up to a sequence divergence of 50 percent, paralogues are less subjected to conservatory forces than orthologues. It must, however, be taken into account that the paralogues for our study were exclusively from human, whereas the orthologues were from a large variety of species.

The analysis that we have performed relies upon the concept of the molecular clock [6], i .e. the more time has elapsed the more amino acid exchanges have accumulated in a protein sequence such that the number of accumulated exchanges can be used to measure the time that has elapsed since two proteins have diverged. Although it has turned out that there is no universal molecular clock [24], but rather that various molecular clocks run at different paces across species, proteins and times, we think that the concept is applicable for our purpose since we constructed the substitution matrices for each degree of divergence from a wide variety of different proteins and species. We did not try to calculate precise time periods that have elapsed since two proteins have diverged, but we do claim that more divergent protein pairs are likely to have diverged earlier than less divergent protein pairs.

Our analysis relies furthermore upon the correctness of the pairwise protein alignments that serve as our data basis. The Pfam-A collection of multiple sequence alignments are produced by first establishing a hand-curated seed alignment of a couple of protein sequences from which a profile Hidden Markov Model is built which again is used to search for homologous sequences in primary sequence data bases. Finally the alignments are checked again manually (P Coggill, personal communication). In such a way the risk to sample non-homologous sequences which could happen by applying an inappropriate substitution matrix is minimized. Since we sampled our pairwise alignments from multiple alignments which consist typically of dozens of sequences (median ∼80) and which can therefore be considered as stable, we are quite confident that the overwhelming majority of the alignments we used are of good quality.

When we spoke about the genetic code in this work we tacitly assumed the universality of the genetic code. There are, however, organisms using slight variants of the standard genetic code. To date, about a dozen of these exceptions are known. We have not checked explicitly if the proteins we used were all translated using the standard genetic code but a few exceptions would certainly not affect our results. Even for non-standard codes the vast majority of assignments between codons and amino acids would be the same as in the standard genetic code.

A limitation of our study is that we did not distinguish between the two directions of an amino acid substitution whose rates are priori not identical. The reason for this was that the construction of such (in general asymmetric) substitution matrices requires the inclusion of an outgroup sequence in the protein sequence alignment. Whereas this was possible for almost all orthologous alignments it was only possible for a fraction of the paralogous alignments. We repeated the correlation analysis with the asymmetric substitution matrices from orthologues and obtained essentially the same results.

It was our endeavor to explain the observed amino acid exchange frequencies for the whole range of protein sequence divergence by means of fundamental structures or forces, like the structure of the genetic code or the physico-chemical forces that maintain an amino acid's character. We therefore had to keep our model somewhat simplistic. It is, for example, known that transversions (interchanges between a purine and a pyrimidine) are much rarer than transitions (interchanges between purines or between pyrimidines). We defined the weighted codon distance by attributing the score 2 to transversions and the score 1 to transitions, such that codon pairs involving transversions are assigned greater distances. This modification, however, did not alter the correlation coefficients between the substitution matrices and the codon distance shown in Figs 4–6. More refined models for codon distance could, for example, also incorporate the codon usage. This will, however, be a much more complex analysis because of the dependence of codon usage on the species and remains as a future challenge.
